# Variability in the Drug Response of M_4_ Muscarinic Receptor Knockout Mice During Day and Night Time

**DOI:** 10.3389/fphar.2019.00237

**Published:** 2019-03-18

**Authors:** Paulina Valuskova, Vladimir Riljak, Sandor T. Forczek, Vladimir Farar, Jaromir Myslivecek

**Affiliations:** ^1^Institute of Physiology, First Faculty of Medicine, Charles University, Prague, Czechia; ^2^Isotope Laboratory, Institute of Experimental Botany, Academy of Sciences of the Czech Republic, Prague, Czechia

**Keywords:** M_4_ muscarinic receptor, biorhythm, motor activity, temperature, scopolamine, oxotremorine, cocaine, open field

## Abstract

Mice are nocturnal animals. Surprisingly, the majority of physiological/pharmacological studies are performed in the morning, i.e., in the non-active phase of their diurnal cycle. We have shown recently that female (not male) mice lacking the M_4_ muscarinic receptors (MR, M_4_KO) did not differ substantially in locomotor activity from their wild-type counterparts (C57Bl/6Tac) during the inactive period. Increased locomotion has been shown in the active phase of their diurnal cycle. We compared the effects of scopolamine, oxotremorine, and cocaine on locomotor response, hypothermia and spontaneous behavior in the open field arena in the morning (9:00 AM) and in the evening (9:00 PM) in WT and in C57Bl/6NTac mice lacking the M_4_ MR. Furthermore, we also studied morning vs. evening densities of muscarinic, GABA_A_, D_1_-like, D_2_-like, NMDA and kainate receptors using autoradiography in the motor, somatosensory and visual cortex and in the striatum, thalamus, hippocampus, pons, and medulla oblongata. At 9:00 AM, scopolamine induced an increase in motor activity in WT and in M_4_KO, yet no significant increase was observed at 9:00 PM. Oxotremorine induced hypothermic effects in both WT and M_4_KO. Hypothermic effects were more evident in WT than in M_4_KO. Hypothermia in both cases was more pronounced at 9:00 AM than at 9:00 PM. Cocaine increased motor activity when compared to saline. There was no difference in behavior in the open field between WT and M_4_KO when tested at 9:00 AM; however, at 9:00 PM, activity of M_4_KO was doubled in comparison to that of WT. Both WT and KO animals spent less time climbing in their active phase. Autoradiography revealed no significant morning vs. evening difference. Altogether, our results indicate the necessity of comparing morning vs. evening drug effects

## Introduction

Mice are nocturnal animals (Roedel et al., 2006). Nevertheless, the majority of physiological/pharmacological studies are performed during the morning. In other words, experiments are conducted in their non-active phase of the diurnal cycle. The question is obvious: could the results of such physiological/pharmacological experiments be affected by this discrepancy?

We have shown recently (Valuskova et al., 2018b) that female mice lacking the M_4_ muscarinic receptors (MR, M_4_KO) did not differ substantially in locomotor activity from their wild-type counterparts (C57Bl/6Tac) during the light period. In contrast, in the dark phase (active phase of the diurnal cycle in mice), the M_4_KO mice exhibited changes in various parameters; briefly, their activity was higher. Interestingly, these differences were sex related and were not observed in males.

Even the molecules involved in cholinergic signaling pathways have been shown to reveal a certain level of circadian rhythmicity. A circadian rhythmicity has been described for acetylcholine release, acetylcholinesterase (AChE) and choline acetyltransferase activity (ChAT) (Hut and Van der Zee, 2011), and MR (Bina et al., 1998; Hut and Van der Zee, 2011). Acetylcholine release peaks in the active phase of the diurnal cycle, and the same has been described for the activity of ChAT. In contrast, MR and AChE levels peak in the inactive phase of the diurnal cycle. Circadian rhythmicity also exists in the plasma and liver enzyme activity of AChE and butyrylcholinesterase (BuChE), and this rhythm can be affected by sex hormones (Alves-Amaral et al., 2010). While plasma and liver AChE activity showed no sex differences and were not influenced by castration, BuChE plasma activity was higher in females, and this sex difference was abolished by castration. Another important molecule involved in acetylcholine signaling pathways with circadian rhythmicity is vesicular acetylcholine transporter (VAChT) (Castillo-Ruiz and Nunez, 2007).

Scopolamine is a muscarinic antagonist widely used for its effect on locomotor activity. An increase in locomotion induced by scopolamine has been described in many rodent models (Thornburg and Moore, 1973; Anisman and Cygan, 1975; Bymaster et al., 1993; Farar et al., 2012). Oxotremorine is a muscarinic agonist with strong hypothermic effects and, similarly to scopolamine, has long been used in animal research (Lomax and Jenden, 1966; Gomeza et al., 1999; Bymaster et al., 2001; Farar et al., 2012). Cocaine represents a drug with multiple effects on various transporters (dopamine, serotonin, noradrenaline). The balance between dopamine and acetylcholine levels affects motor activity. Thus, if cocaine affects dopamine transporter (DAT), then cocaine affects dopamine level. Therefore, DAT is the key component (Giros et al., 1996; Thomsen et al., 2009; Sullivan et al., 2013; Vaughan and Foster, 2013) changing dopamine, and (because of dopamine-acetylcholine balance) acetylcholine levels. Acetylcholine levels can affect the MR density. This is the pathway connecting changes in dopamine and muscarinic receptors. Another molecule, that is influenced by cocaine action is D_1_ dopaminergic receptor (Caine et al., 2007). In addition to the dopaminergic system, an increasing body of evidence suggests an important role for the central cholinergic system (particularly cholinergic interneurons) located in the nucleus accumbens (NAc) in the regulation of cocaine action (Hikida et al., 2001; Crespo et al., 2006; Witten et al., 2010; Dencker et al., 2012). One of the most commonly used tests for behavior classification and analysis is the open field test. This test was developed by Calvin S. Hall in the 30s of the 20th century (Stanford, 2007) to test the “emotionality” of the animals by the defecation score. However, as reviewed by Stanford (2007), there are some significant shortcomings. In general, the open field test examines only general locomotor activity levels and willingness to explore (often considered as a sign of anxiety) in rodents. Because the open field test is one of the most commonly used behavioral tests, we have chosen to use it to further test locomotor activity.

According to our knowledge, the evidence for morning vs. evening muscarinic effects in locomotor activity regulation, thermoregulation and behavior tests is missing. There are no reference data/results for drugs such as scopolamine, oxotremorine, and cocaine.

We therefore performed a series of experiments in which we compared the effects of scopolamine, oxotremorine, and cocaine on locomotor response or hypothermia in the morning (9:00 AM) and in the evening (9:00 PM) in two mouse strains: WT animals (C57Bl/6Tac) and mice lacking the M_4_ MR (M_4_KO), in which increased locomotion has been shown in the active (dark) phase of their diurnal cycle (Valuskova et al., 2018b). Only females were used, as no differences in locomotor activity related to the biorhythm were observed between M_4_ KO and WT males. In addition, we also tested the same animal lines in the open field with the major aim of evaluating their reaction to the novel environment and comparing their responses in the morning and in the evening. We hypothesized that the morning and evening responses would differ. Observed differences in these tests would indicate that the common morning testing does not necessarily reflect the real drug effects.

The motor coordination is the result of many receptor/neurotransmitter systems interactions. GABA, glutamate, and acetylcholine levels in the striatum are mutually interconnected. Similar interactions can be found in other brain structures. Thus, we have decided to map neurotransmitter receptors to further elucidate the relationship between the abovementioned functional effects of cocaine, scopolamine, and oxotremorine and particular receptor systems. We tested the densities of muscarinic, GABA_A_, D_1_-like, D_2_-like, NMDA and kainate receptors using the autoradiography technique. Various structures were inspected, including the motor, somatosensory and visual cortex (VisCx), striatum, NAc, thalamus (TH), hippocampus (Hipp) (dorsal hippocampus, dentate gyrus, CA1, CA3 area), olfactory tubercle, pons, and the medulla oblongata.

## Materials and Methods

### Animals

Mice lacking the M_4_ muscarinic receptors were generated in the Wess laboratory (Gomeza et al., 1999), bred in our animal facility (Prague, Czechia) and maintained for at least 12 generations on the genetic background C57Bl/6Tac. Animals were treated in accordance with the legislature of the Czech Republic and the EU legislature [European Convention for the Protection of Vertebrate Animals used for Experimental and other Scientific Purposes (Council of Europe N^o^ 123, Strasbourg 1985)], and the experimental protocol was approved by the Committee for the Protection of Experimental Animals of the First Medical Faculty, Charles University, Prague and by the Ministry of Education of the Czech Republic under N^o^ MSMT-2409/2017-3. The wild-type line was the same as the background line in KO animals, i.e., C57Bl/6Tac. We studied fully backcrossed (12 generations) muscarinic M_4_^-/-^ (M_4_KO) and M_4_^+/+^ littermates. The animals were maintained under controlled environmental conditions (12/12 h light/dark cycle, 22 ± 1°C, light on at 6:00 AM). Food and water were available *ad libitum*. A total of 121 females (weighing 20–26 g, aged 3–6 months, 5–10 animals per group) were included in the experiment: 62 M_4_KO animals and 59 WT counterparts were used in the study. The animals were used for one behavioral test only (the tests were performed on different animals). The animals were first used in the open field test and then they were treated with drugs. The autoradiography was performed on control animals receiving saline only at least 2 weeks before. Females were housed separately from males and thus were synchronized in their estrus cycle (anestrus, confirmed by lavage and light microscopy). Prior to the experiments, the mice were genotyped, and only homozygotes were used in the study.

### Telemetry

To continuously measure temperature and locomotor activity, we employed a telemetric apparatus. The telemetry system used in this study was commercially available from Mini Mitter (Starr Life Sciences Corp., Oakmont, PA, United States, originally from Respironics, Andover, MA, United States). Receivers were connected in series and connected directly to the PC into a single computer port allowing the determination of all collected parameters. The transponders (E-Mitter, G2, length 15.5 mm, 1.1 g) were implanted in the peritoneal cavity under anesthesia (Zoletil^®^ 100, Rometar^®^ 2% 5:1, diluted 10 times, 3.2 ml.kg^-1^). During the surgery, the mice were kept on a thermostable pad. After the implantation, the mice were left undisturbed at least 11 days for recovery from the surgery. Body temperature and activity were acquired directly from the transponders in the sample period over seven consecutive days in which the animals were not disturbed. Data were sampled every 60 s. VitalView software was used for data acquisition and data processing.

### Application of Drugs

To compare the effects of morning vs. evening administration of three centrally acting motor activity/temperature drugs, scopolamine (5 mg/kg, s.c.), cocaine (20 mg/kg, s.c.) or oxotremorine (0.2 mg/kg, s.c.) were administered at 9:00 AM or at 9:00 PM, i.e., in a sufficient interval (after 3 h) from light change (light on at 6:00 AM, light off at 6:00 PM). Used doses were determined by pilot experiments. All experiments, performed at 9:00 PM, were carried out under red light to respect the natural light/dark behavioral activity pattern and biological rhythms. Drugs were freshly diluted in saline on the day of administration. Controls received saline only. Motor activity and temperature were monitored using a telemetry system as described above. Motor activity was observed 60 min after scopolamine or cocaine application. Oxotremorine effects were observed 360 min after application, and maximal drop of temperature, recovery time to baseline temperature and area under the curve were established. For the recovery time to baseline temperature 1-h average values were considered as baseline. If the temperature return to the initial values remained stable for the next 10 min, it was considered as point of return. The initial treatment of data was performed using VitalView software; later, the data were statistically analyzed using GraphPad Prism (San Diego, CA, United States).

### Open Field

To assess the response of the animals to a novel environment, we used the open field test. We compared the behavior of the animals at 9:00 AM and 9:00 PM. The mice were placed into a transparent plastic box (38 × 22 × 15 cm), and data on motor activity were recorded for 60 min using a telemetry system. Then, the mice were placed in their original cages. The experiments, performed at 9:00 PM, were carried out under red light. Data were sampled every 60 s. The initial treatment of data was performed using VitalView software; later, the data were statistically analyzed using GraphPad Prism (San Diego, CA, United States).

### Automated Detection System in the Open Field: Laboras Apparatus

Spontaneous behavior was also analyzed in a special open field arena. Animals were weighed, marked and placed in a Laboras apparatus (Metris B.V., Netherlands) to monitor their behavior for 10 min. After each session, animals were returned to their home cages. Laboras is an automated system for continuous behavior tracking and analysis. Mechanical vibrations generated by an animal (locomotion, rearing, grooming, etc.) are transformed into an electrical signal. Such signals are processed, classified and compared with the predetermined characteristic patterns of Laboras software (Van de Weerd et al., 2001). Collected data describe different aspects of the animal locomotion. The following parameters were analyzed: locomotion (duration and counts), rearing (duration and counts), distance traveled, and climbing (duration and counts). Each measured behavioral parameter was evaluated separately using GraphPad Prism software.

### Receptor Autoradiography

#### Tissue Preparation

For receptor determination, autoradiography was performed in several brain areas [motor cortex (MOCx), somatosensory cortex (SSCx), VisCx, striatum (caudate-putamen, CPu), NAc, TH, Hipp and its specific areas CA1, CA3 and dentate gyrus (DG), olfactory tubercle (OT), pons (Pons) and medulla oblongata (MY)] on sagittal brain sections of M_4_KO mice and their WT littermates as described previously (Valuskova et al., 2018a,b).

#### Autoradiography of Receptors

Muscarinic receptors were detected using ^3^H-QNB radioligand binding. For GABA_A_ receptor detection, we used ^3^H-muscimol. D_1_-like dopamine receptors were detected using ^3^H-SCH 23390. D_2_-like dopamine receptors were detected using ^3^H-spiperone. NMDA receptors were detected using ^3^H-CGP 39653, and kainate receptors were detected by ^3^H-kainate radioligand binding. Autoradiography was performed as described previously in detail (Farar et al., 2012). Autoradiography receptor determination was performed on mice sacrificed at 9:00 AM and at 9:00 PM.

### Statistical Analysis

For the comparison of multiple groups, we used three-way ANOVA or two-way ANOVA with *post hoc* Tukey’s corrections. For comparison of ranks, Mann–Whitney test was used. Values of *p* < 0.05 were considered as significant.

## Results

### Scopolamine Administration

**FIGURE 1:**
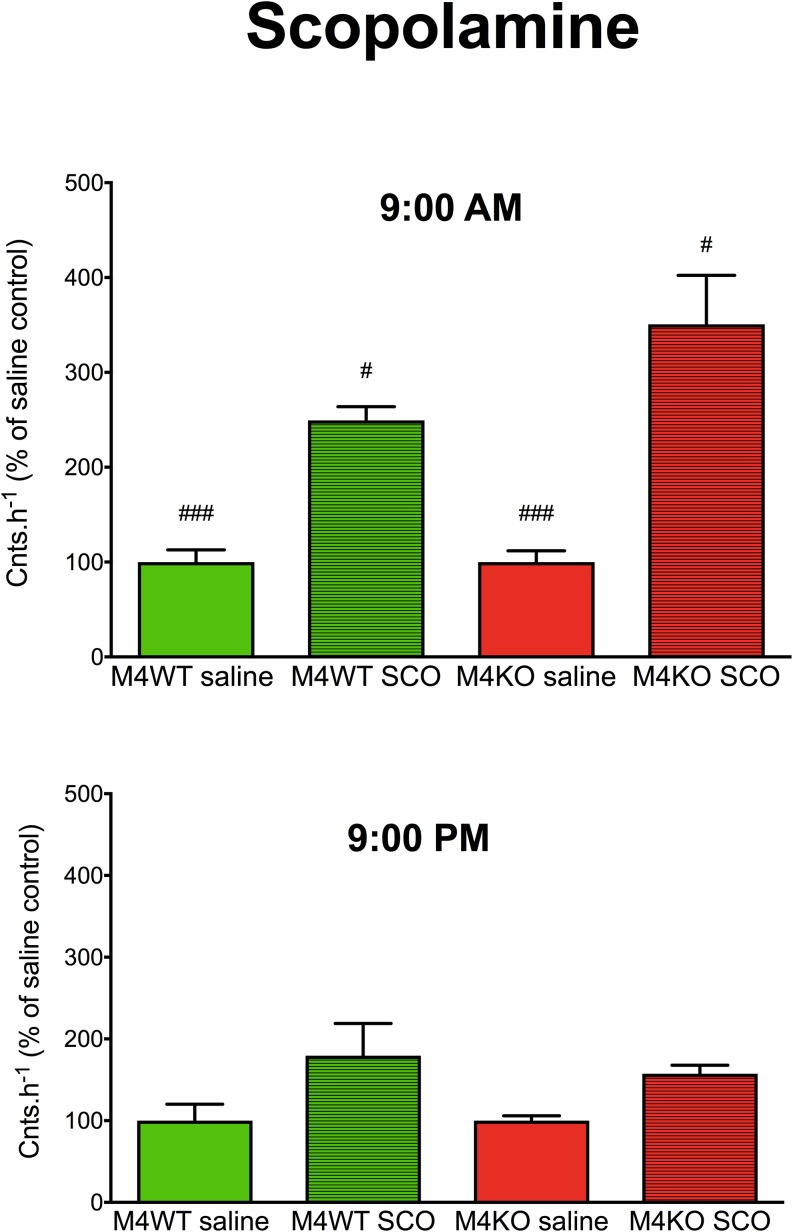
The effects of scopolamine (SCO) on locomotor activity in WT (M_4_WT) and KO (M_4_KO) mice at 9:00 AM (top) and 9:00 PM (bottom). Control mice were treated with physiological solution (saline). Ordinate: counts per hour expressed as % of activity in saline injected mice. ^#^*p* < 0.05, ^###^*p* < 0.001, difference from PM.

A three-way ANOVA (genotype: wild type/knockout; drug: saline/scopolamine; time: AM/PM) revealed significant main effect (Figure 1) of the applied drug (F_DFn,DFd_, where DFn is degrees of freedom numerator, and DFd is degrees of freedom denominator: *F*_1,20_ = 41.90, *p* < 0.001) and main effect of time (*F*_1,20_ = 10.05, *p* = 0.005) and no effect of genotype (*F*_1,20_ = 0.91, *p* = 0.35). There was also significant interaction between genotype and time (*F*_1,20_ = 10.05, *p* = 0.005, both saline and scopolamine differed in AM vs. PM effects, *p* < 0.001, *p* = 0.03 for saline and scopolamine, respectively). Other interactions were non-significant (genotype × drug: *F*_1,20_ = 0.91, *p* = 0.35; genotype × time: *F*_1,20_ = 2.21, *p* = 0.15; genotype × drug × time: *F*_1,20_ = 2.21, *p* = 0.15).

### Oxotremorine Administration

**FIGURE 2:**
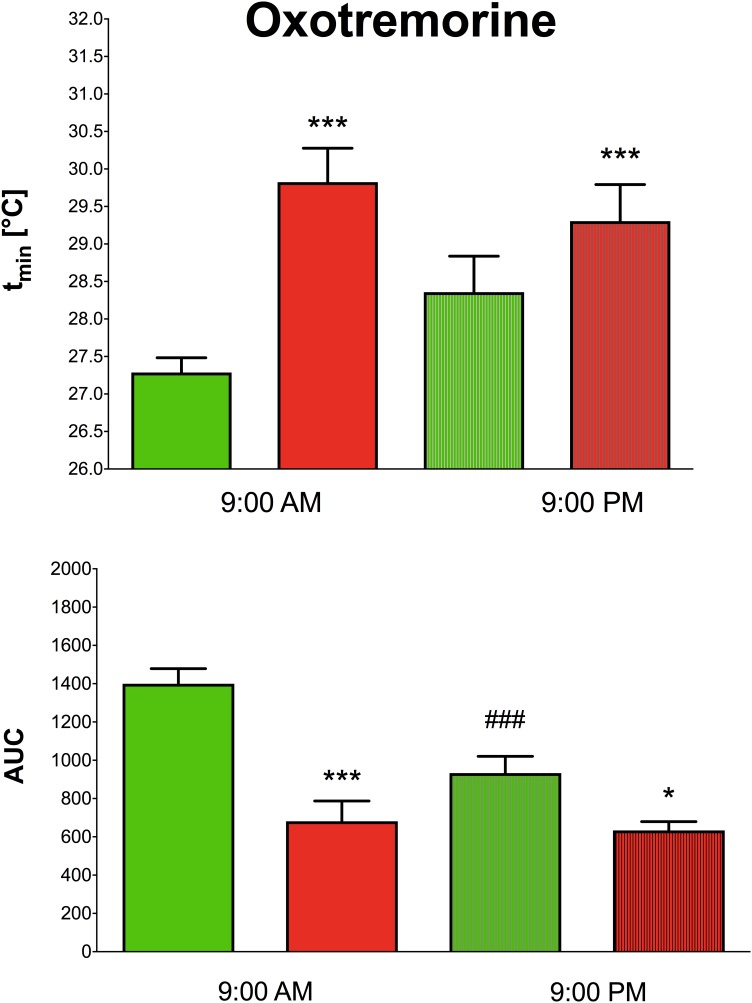
The effects of oxotremorine on the maximal drop in temperature in WT (green columns) and KO (red columns) at 9:00 AM and at 9:00 PM (top). Ordinate: *t*_min_ [°C] minimal temperature. The effects of oxotremorine on the area under the curve (AUC) in WT (green columns) and KO (red columns) mice at 9:00 AM and at 9:00 PM (bottom). ^∗^*p* < 0.05, ^∗∗∗^*p* < 0.001, difference from WT mice, ^###^*p* < 0.001, difference from the same strain at 9:00 AM.

**FIGURE 3:**
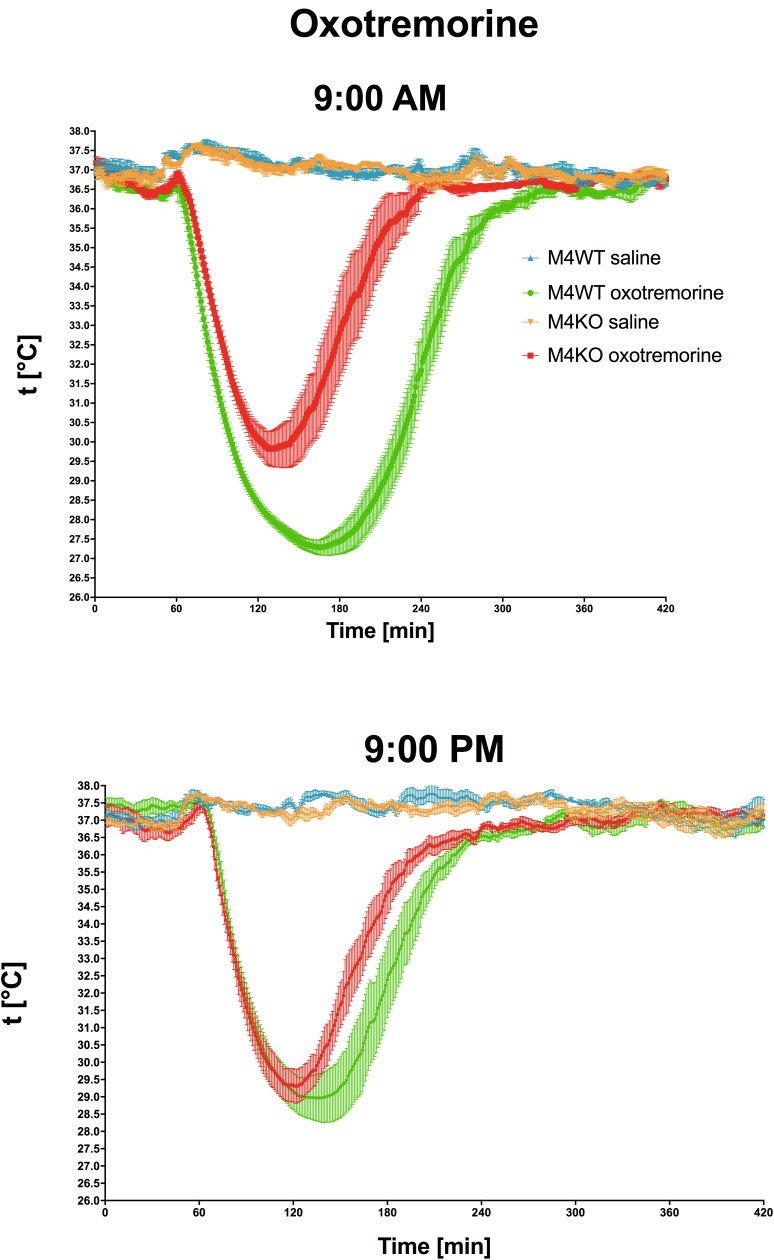
The effects of oxotremorine/saline on temperature 360 min after oxotremorine application. The temperature was also monitored 60 min before drug application. Ordinate: *t* [°C], temperature. Abscise: time [min]. Top: the effects at 9:00 AM (oxotremorine/saline application); bottom: the effects at 9:00 PM (oxotremorine/saline application). No changes in temperature were recorded after saline application.

Oxotremorine application led to hypothermic effects in both WT and KO animals. The maximal drop in temperature (Figure 2 top, expressed as *t*_min,_ minimal temperature measured) was greater (two-way ANOVA, genotype: *F*_1,15_ = 17.28, *p* < 0.001; time: *F*_1,15_ = 0.43, *p* = 0.52; interaction genotype–time: *F*_1,15_ = 3.60, *p* = 0.08) in WT animals than in KO animals (9.29°C vs. 6.88°C, respectively) at 9:00 AM, and 9:00 PM (8.22°C vs. 7.4°C, respectively). The recovery time to baseline temperature (Figure 3) was similar in WT and KO animals when tested at 9:00 PM. Recovery lasted 306 min in KO animals, and it took 411 min in WT animals when measured at 9:00 AM (Mann–Whitney test of ranks, *p* = 0.008). The area under the curve (Figure 2 bottom and also visible on Figure 3) was greater (two-way ANOVA, genotype: *F*_1,16_ = 38.31, *p* < 0.001; time: *F*_1,16_ = 9.78, *p* = 0.006; interaction genotype–time: *F*_1,16_ = 6.49, *p* = 0.02) in WT animals at 9:00 AM than in KO animals (it was approximately doubled) and also differ at 9:00 PM (was decreased in KO animals to 68% of WT value). Moreover, the AUC in WT animals at 9:00 PM was reduced to 67% of the value at 9:00 AM.

### Cocaine Administration

**FIGURE 4:**
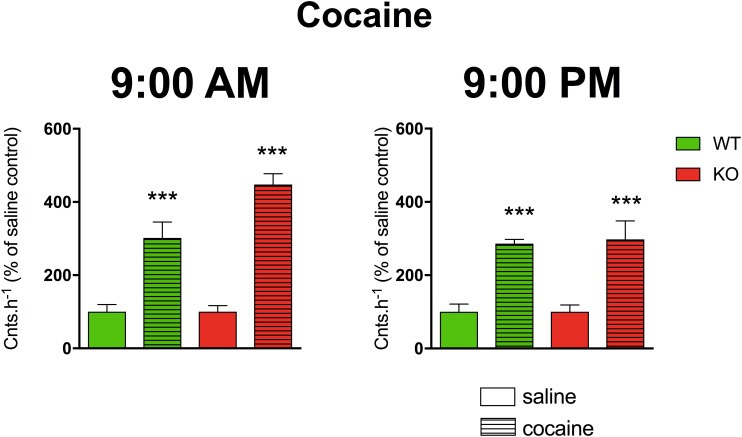
The effects of cocaine on locomotor activity in WT and KO mice at 9:00 AM (left) and 9:00 PM (right). Control mice were treated with physiological solution (saline), see legend in the figure. Ordinate: counts per hour expressed as % of activity in saline-injected mice. ^∗∗∗^*p* < 0.001, difference from saline-injected mice.

A three-way ANOVA (genotype: wild type/knockout; drug: saline/cocaine; time: AM/PM) revealed significant main effects of the applied drug (*F*_1,21_ = 85.29, *p* < 0.001). No effect of genotype (*F*_1,21_ = 2.44, *p* = 0.13) and time (*F*_1,24_ = 2.68, *p* = 0.12) was revealed. There was no significant interaction between all combinations of factors (genotype and time (*F*_1,21_ = 1.76, *p* = 0.20), genotype and drug (*F*_1,21_ = 2.44, *p* = 0.13), drug and time (*F*_1,21_ = 2.68, *p* = 0.12) and interaction of all three factors revealed *F*_1,21_ = 1.76, *p* = 0.20). Cocaine increased motor activity (Figure 4) when compared to saline (*p* < 0.001).

### Open Field Test

**FIGURE 5:**
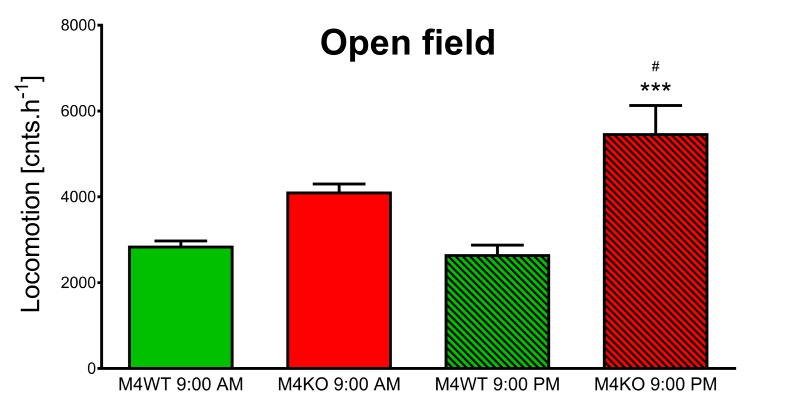
Exploration of the novel area in the open field. The differences in WT (M_4_WT) and KO (M_4_KO) mice at 9:00 AM (left) and 9:00 PM (right). ^∗∗∗^*p* < 0.001, difference from WT mice; ^#^*p* < 0.05, difference from mice tested at 9:00 AM.

Two-way ANOVA revealed the effect of genotype (*F*_1,65_ = 33.62, *p* < 0.001) and interaction between genotype and time (*F*_1,65_ = 4.91, *p* = 0.03). *Post hoc* analysis showed no differences in the locomotor activity in the novel environment (1-h open field test) between WT and KO animals when tested at 9:00 AM (Figure 5, *p* = 0.06). In contrast, at 9:00 PM, the activity in KO animals was doubled in comparison to that in WT animals (*p* < 0.001). Moreover, the activity of KO animals at 9:00 PM was by 42% higher than the activity of KO animals tested at 9.00 AM (*p* = 0.04).

### Detailed Behavior Analysis Using the Laboras Apparatus

**FIGURE 6:**
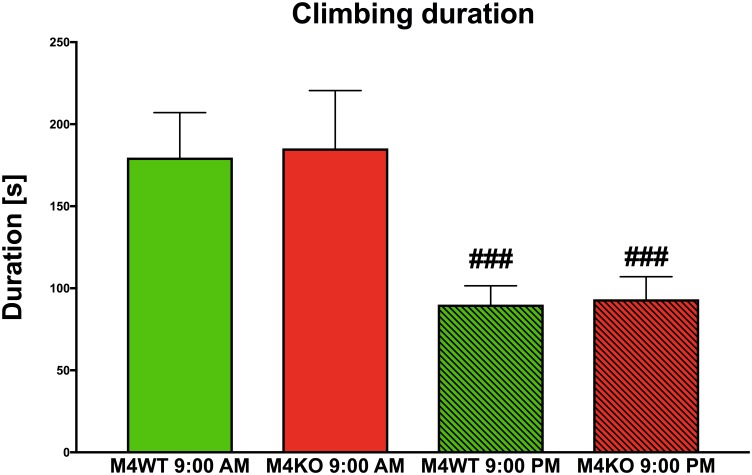
The duration of climbing in the Laboras apparatus (more detailed open field test). The differences in WT (M_4_WT) and KO (M_4_KO) mice at 9:00 AM (left) and 9:00 PM (right). ^###^*p* < 0.001, difference from mice tested at 9:00 AM.

We found no difference in 10-min locomotion (duration and counts), rearing (duration and counts), or distance traveled (all data not shown) at 9:00 AM vs. 9:00 PM. However, significant differences were found in climbing duration (see Figure 6). Two-way ANOVA revealed the effect of time (*F*_1,30_ = 15.50, *p* < 0.001) and no effect both in genotype (*F*_1,30_ = 0.04, *p* = 0.85) and interaction between genotype and time (*F*_1,30_ = 0.002, *p* = 0.96). *Post hoc* analysis showed that both WT and KO animals spent less time climbing in their active phase (9:00 PM, *p* < 0.001).

### Receptor Autoradiography

#### Muscarinic Receptors

**FIGURE 7:**
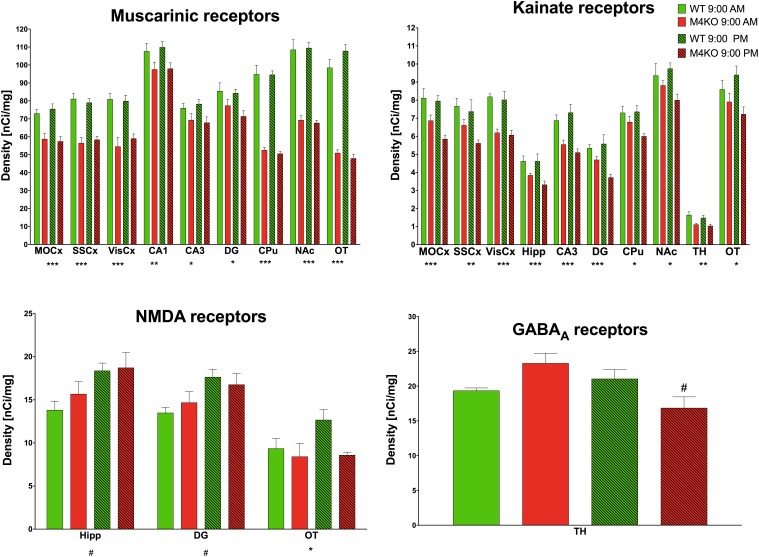
Changes in receptor densities (muscarinic receptors, kainate receptors, NMDA receptors, GABA_A_ receptors) revealed by autoradiography. Only significant results are shown. Motor cortex (MOCx), somatosensory cortex (SSCx), visual cortex (VisCx), striatum (caudate-putamen, CPu), nucleus accumbens (NAc), thalamus (TH), hippocampus (Hipp) and its specific areas CA3 and dentate gyrus (DG), and olfactory tubercle (OT). ^∗^*p* < 0.05, ^∗∗^*p* < 0.01, and ^∗∗∗^*p* < 0.001, difference from WT mice; ^#^*p* < 0.05, difference from mice tested at 9:00 AM. See legend for group explanation.

Muscarinic receptor densities (all binding data are in Supplementary Table S1 and examples of binding are in Supplementary Figure S1) in the MOCx (see Figure 7, two-way ANOVA; genotype: *F*_1,19_ = 36.54, *p* < 0.001; time: *F*_1,19_ = 0.05, *p* = 0.83, interaction: *F*_1,19_ = 0.52, *p* = 0.48) were lower in the KO animals than in the WT animals. Similar decrease was found in the SSCx (two-way ANOVA; genotype: *F*_1,19_ = 72.12, *p* < 0.001; time: *F*_1,19_ = 0.001, *p* = 0.97, interaction: *F*_1,19_ = 0.54, *p* = 0.47) and in the VisCx (two-way ANOVA; genotype: *F*_1,19_ = 40.15, *p* < 0.001; time: *F*_1,19_ = 0.22, *p* = 0.64, interaction: *F*_1,19_ = 0.52, *p* = 0.48). There was also decrease in MR in the dentate gyrus (two-way ANOVA; genotype: *F*_1,19_ = 9.13, *p* = 0.007; time: *F*_1,19_ = 1.05, *p* = 0.32, interaction: *F*_1,19_ = 0.49, *p* = 0.49). The decreases in MR in the CA1 area (two-way ANOVA; genotype: *F*_1,19_ = 8.74, *p* = 0.008; time: *F*_1,19_ = 0.13, *p* = 0.72, interaction: *F*_1,19_ = 0.06, *p* = 0.81), in the CA3 area (two-way ANOVA; genotype: *F*_1,19_ = 7.77, *p* = 0.012; time: *F*_1,19_ = 0.02, *p* = 0.89, interaction: *F*_1,19_ = 0.38, *p* = 0.54), in OT (two-way ANOVA; genotype: *F*_1,19_ = 284.67, *p* < 0.0001; time: *F*_1,19_ = 1.00, *p* = 0.33, interaction: *F*_1,19_ = 3.79, *p* = 0.07), in the striatum (two-way ANOVA; genotype: *F*_1,19_ = 214.0, *p* < 0.001; time: *F*_1,19_ = 0.15, *p* = 0.71, interaction: *F*_1,19_ = 0.09, *p* = 0.77), and in NAc (two-way ANOVA; genotype: *F*_1,19_ = 133.63, *p* < 0.001; time: *F*_1,19_ = 0.01, *p* = 0.93, interaction: *F*_1,19_ = 0.14, *p* = 0.71) were observed.

#### GABA_A_ Receptors

The TH was the only brain area where a difference (two-way ANOVA; genotype: *F*_1,12_ = 0.26, *p* = 0.62; time: *F*_1,12_ = 1.19, *p* = 0.30, interaction: *F*_1,12_ = 5.34, *p* = 0.04) in GABA_A_ receptor density was detected. M_4_ KO animals showed a 25% decrease in GABA_A_ receptor density at 9:00 PM when compared to the density at 9:00 AM.

#### D_1_-Like and D_2_-Like Dopamine Receptors

No brain area showed differences in any tested dopamine receptor subclass.

#### NMDA Receptors

The NMDA receptor density in the Hipp revealed an increase when comparing AM and PM values (two-way ANOVA; genotype: *F*_1,10_ = 0.71, *p* = 0.42; time: *F*_1,10_ = 8.33, *p* = 0.02, interaction: *F*_1,0_ = 0.33, *p* = 0.58). Similar increase was seen in DG (two-way ANOVA; genotype: *F*_1,10_ = 0.02, *p* = 0.88; time: *F*_1,10_ = 8.74, *p* = 0.01, interaction: *F*_1,10_ = 0.97, *p* = 0.35). In the olfactory tubercle, the density of NMDA receptors in M_4_ KO animals was lower than that in their WT counterparts (two-way ANOVA; genotype: *F*_1,8_ = 7.70, *p* = 0.02; time: *F*_1,8_ = 0.39, *p* = 0.55, interaction: *F*_1,8_ = 3.54, *p* = 0.10).

#### Kainate Receptors

Lower density of kainate receptors in the MOCx (two-way ANOVA; genotype: *F*_1,9_ = 22.25, *p* = 0.001; time: *F*_1,9_ = 2.71, *p* = 0.13, interaction: *F*_1,9_ = 1.47, *p* = 0.26) was found when comparing KO and WT animals. There was also a decrease when comparing KO and WT animals in the SSCx (two-way ANOVA; genotype: *F*_1,9_ = 10.98, *p* = 0.009; time: *F*_1,9_ = 2.30, *p* = 0.16, interaction: *F*_1,9_ = 0.68, *p* = 0.43), in the VisCx (two-way ANOVA; genotype: *F*_1,9_ = 47.44, *p* < 0.001; time: *F*_1,9_ = 2.30, *p* = 0.16, interaction: *F*_1,9_ = 0.68, *p* = 0.43), in the Hipp (two-way ANOVA; genotype: *F*_1,9_ = 32.83, *p* < 0.001; time: *F*_1,9_< 0.001, *p* = 0.98, interaction: *F*_1,9_ = 2.04, *p* = 0.19), in the CA3 area (two-way ANOVA; genotype: *F*_1,10_ = 22.39, *p* < 0.001; time: *F*_1,10_ = 0.19, *p* = 0.67, interaction: *F*_1,10_ = 1.47, *p* = 0.25), in the dentate gyrus (two-way ANOVA; genotype: *F*_1,9_ = 19.56, *p* < 0.001; time: *F*_1,9_ = 1.73, *p* = 0.22, interaction: *F*_1,9_ = 4.61, *p* = 0.06), in CPu (two-way ANOVA; genotype: *F*_1,10_ = 8.25, *p* = 0.017; time: *F*_1,10_ = 1.27, *p* = 0.29, interaction: *F*_1,10_ = 1.70, *p* = 0.22), in NAc (two-way ANOVA; genotype: *F*_1,10_ = 8.25, *p* = 0.017; time: *F*_1,10_ = 1.27, *p* = 0.29, interaction: *F*_1,10_ = 1.70, *p* = 0.22), in the TH (two-way ANOVA; genotype: *F*_1,10_ = 20.03, *p* = 0.001; time: *F*_1,10_ = 1.26, *p* = 0.27, interaction: *F*_1,10_ = 0.22, *p* = 0.65), and in OT (two-way ANOVA; genotype: *F*_1,10_ = 8.83, *p* = 0.014; time: *F*_1,10_ = 0.02, *p* = 0.91, interaction: *F*_1,10_ = 2.37, *p* = 0.16),

## Discussion

We have shown here that the effects of drugs affecting the MR (oxotremorine, scopolamine), and activity in a novel environment differ substantially depending on the time of day. We observed more pronounced drug effects in the morning (9:00 AM) than in the evening (9:00 PM). In contrast, the open field activity was increased in the active phase (at 9:00 PM) of the animal. We also showed that the drug response and behavioral pattern could differ dramatically between WT and KO animals if tested in the morning or in the evening. Therefore, we conclude that testing in the inactive or resting phase of the diurnal cycle in nocturnal animals can significantly distort the effect of drugs tested in the natural active phase of the diurnal cycle of the animal.

Although chronopharmacology and chronotherapeutics (Dallmann et al., 2014; Ballesta et al., 2017) represent highly interesting and researched areas in pharmacology/pharmacotherapeutics, the evidence for animal muscarinic chronopharmacology is missing. A realistic description of animal muscarinic chronopharmacology in the context of muscarinic drug usage in locomotor activity regulation, thermoregulation and behavior tests is missing. To the best of our knowledge, there are no reference data/results for drugs such as scopolamine, oxotremorine, and cocaine administered in the evening; thus, the results of our study cannot be compared with those of another study. For data on M_4_ MR effects on locomotor response, please see discussion in Valuskova et al. (2018b).

Scopolamine is considered a drug able to increase the intensity of locomotor activity. We observed an increase in locomotor activity both in WT and KO animals in the morning, which is in agreement with previous observations (Thornburg and Moore, 1973). However, when we tested the effects of scopolamine at 9:00 PM, we failed to observe an increase in locomotor activity in WT or KO animals. Therefore, scopolamine administration during the light day phase may significantly distort the results. In the dark (active) phase of the diurnal cycle, the mice did not exert any observable changes in their locomotor activity after scopolamine treatment.

Oxotremorine is used to decrease temperature by activation of muscarinic receptors (Lomax and Jenden, 1966). We found that even if hypothermic effects were present at 9:00 AM as well as at 9:00 PM, the temperature decrease at 9:00 AM in WT mice was more prominent than that observed in KO animals, while no difference between WT and KO was found at 9:00 PM. The recovery time to baseline temperature was similar in WT and KO animals when measured in the evening, while it differed in the morning. The area under the curve describing the course of hypothermia and recovery to baseline temperature in WT animals was approximately doubled than in KO animals at 9:00 AM but did not differ at 9:00 PM. Moreover, the AUC in WT animals at 9:00 PM was reduced to 67% of the value observed at 9:00 AM. These data show clear differences in oxotremorine action in the inactive vs. active phase of the animal diurnal cycle: in this case, the test in the morning showed differences between WT and KO animals, while the test in the naturally active phase showed no differences.

Cocaine is a multiple target drug with a prominent preference for DAT. The striatal balance between dopamine and acetylcholine levels affects motor activity. Thus DAT, affecting dopamine level, can affect acetylcholine level which in turn can affect MR. It is well-established that cocaine, among other effects, increases locomotor activity (Dall et al., 2017). In contrast to scopolamine and oxotremorine, there are some data about the diverse effects of cocaine after morning/evening administration. Diurnal variations in cocaine-conditioned place preference have been observed (Sleipness et al., 2007). The authors hypothesized that such an effect was probably connected with diurnal variation in DAT and tyrosine hydroxylase enzyme, the key component in the catecholamine synthesis pathway (Sleipness et al., 2007). Long-term but not short-term sensitization to cocaine (Sleipness et al., 2005) also reveals certain diurnal variations. These findings are partially in agreement with our data demonstrating the cocaine effects in the active phase.

As mentioned above (Stanford, 2007), the open field test is not represented by one standardized protocol; the parameters of the arena, time of observation, etc. differ from study to study. One of the usual approaches is to test the animals during their active phase of the diurnal cycle. We thus used the open field test as a test of animal reactions to a novel environment, and we compared the results obtained in the morning and in the evening. The references describing the differences between morning vs. evening tests are very rare. A study examining *Octodon degus* has been performed at different times of day (Popoviæ et al., 2009), but this study was comparing young and adult animals and found differences in the mid-day and in the afternoon. Differences between motor activity during the day and night have been described for Sprague-Dawley and Wistar rats (McDermott and Kelly, 2008); however, this study only compared strain differences, and differences in the open field were not found. A diurnal pattern has been found in gerbils (Probst et al., 1987), showing that scent-marking activity, sniffing frequency and locomotor activity in the open field have a clear rhythm with a maximum during the dark period. We also found a diurnal pattern of activity in the open field and were able to show differences between KO and WT mice only in the active phase of the animal cycle. Interestingly, using the Laboras system as another type of open field test, we observed changes in animal climbing. The mice climbed less during their active (dark) phase of the day. Together with the lack of change in locomotion and distance traveled (graphs not shown), these observations show that the behavioral paradigm of habituation differs in the morning and evening. Our results suggest that the time of observation is crucial for the proper interpretation of results. The 10-min open field test shows how an animal copes with a new experimental environment, while the longer test (in our case 1 h) shows the pattern of animal behavior. Again, our test showed morning vs. evening differences.

The changes in QNB binding to muscarinic receptors reflect two aspects of muscarinic receptor number: first, the total number of muscarinic receptors in the specific brain area, and second, the number of M_4_ muscarinic receptors. While the total number of MR is detectable in WT animals (in that case QNB binds to all MR subtypes), in M_4_ KO animals the QNB binding represents all MR except M_4_ MR. Thus, the binding in WT minus binding in KO is the proportion of M_4_ MR in the appropriate brain region. The relative M_4_ MR density was: MoCx: 19% and 24% (morning vs. evening, respectively), SSCx: 30% and 26%, VisCx: 33% and 26%, CA1: 9% and 11%, CA3: 9% and 13%, DG: 9% and 15%, CPu: 45% and 47%, NAc: 36% and 38%, and OT: 48% and 56%. Although there were small differences in the number of receptors in the morning vs. evening, neither was significant (see Results).

The densities of D_1_-like, and D_2_-like were similar in all brain regions tested. Kainate receptors did not generally differ significantly in M_4_KO or WT animals when the morning and evening values were compared. However, in some brain areas, there was a difference between WT and KO animals. These regions were, as follows: MoCx, VisCx, Hipp, CA3, DG, NAc, OT, and TH. These changes suggest mutual interconnection between M_4_ MR and kainate receptors as the brain areas in which receptor changes have occurred were similar.

GABA_A_ and NMDA receptors revealed few differences when comparing morning vs. evening values. Morning/evening difference was seen in NMDA receptors in Hipp and dentate gyrus and in GABA_A_ receptors in the TH. However, it is unlikely that these changes can represent the nature of different muscarinic drug effects when applied at 9:00 AM vs. 9:00 PM. Thus, we can almost certainly exclude the role of all examined receptor systems on differences in drug administration consequences between the morning and evening. One possible explanation is that other steps in intracellular signaling cascades, protein–protein interactions, gentle metabolism modifications or even changes in the neuronal microenvironment (e.g., ion levels, neurotransmitter levels) could cause the observed effects. Of course, mutual interactions between receptor systems are also in play even when there is only a very small change in receptor number.

## Conclusion

Altogether, our results clearly indicate the real necessity to test the effects of drugs in the morning and evening to obtain a realistic picture of their physiological/pharmacological profile. These data only provide evidence for cocaine, scopolamine, and oxotremorine and are moreover limited to WT and M_4_KO female mice. However, our findings are also relevant to WT mice and thus indicate the general applicability of this concept. Therefore, researchers should clearly indicate the time at which any particular test or drug application is performed. Otherwise, the results could be easily distorted and misinterpreted.

## Author Contributions

JM, VR, and VF contributed to the conception and design of the reported studies. PV, VF, and VR conducted all of the experiments. SF, VF, VR, and PV analyzed the data. PV, VR, and VF contributed to the drafting and revision of the manuscript. JM wrote the manuscript in the final form. All authors approved the final version and agreed to be accountable for all aspects of the work.

## Conflict of Interest Statement

The authors declare that the research was conducted in the absence of any commercial or financial relationships that could be construed as a potential conflict of interest.

## Supplementary Material

FIGURE S1 The examples of autoradiography binding pictures (sagittal sections) for specific receptor subtypes. Left column: binding to specific receptor subtype in WT animals. Right column: binding to specific receptor subtype in M_4_ KO animals. First to sixth row: binding to MR, D_1_-like, D_2_-like, GABA_A_, NMDA and kainate receptors, respectively. Bottom: radioactivity standards.

TABLE S1 The autoradiography binding to brain regions for specific receptor subtypes.
