# Supplementary figures and images for: Variability in the Drug Response of M4 Muscarinic Receptor Knockout Mice During Day and Night Time
Source: Front Pharmacol. 2019 Mar 18;10:237. doi: 10.3389/fphar.2019.00237 (PMC6431655; doi:10.3389/fphar.2019.00237)

WT

KO

MR

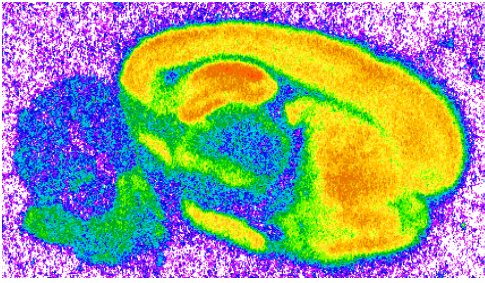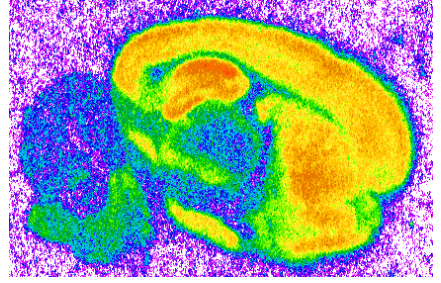

D<sub>1</sub>-like

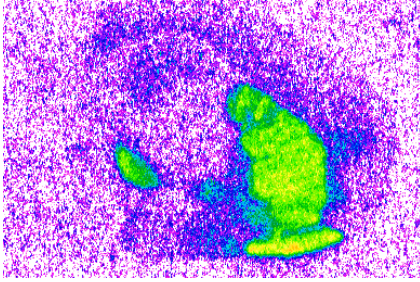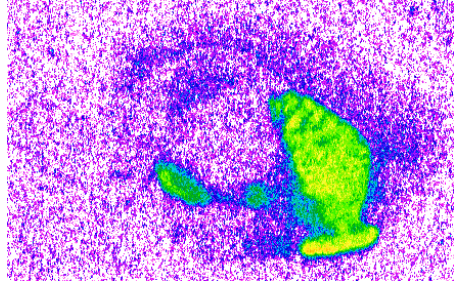

D<sub>2</sub>-like

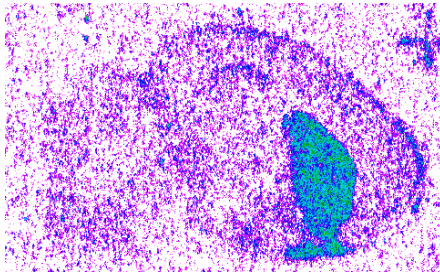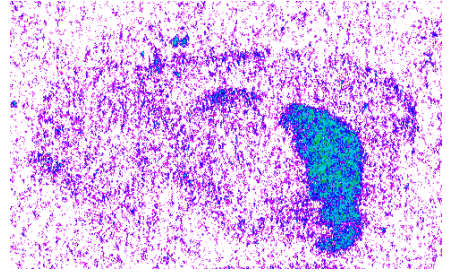

GABA<sub>A</sub>

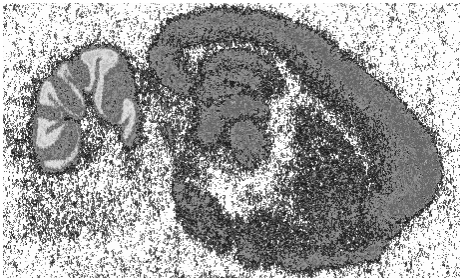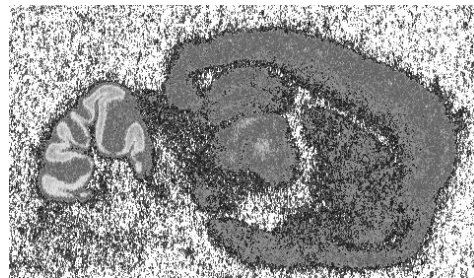

NMDA

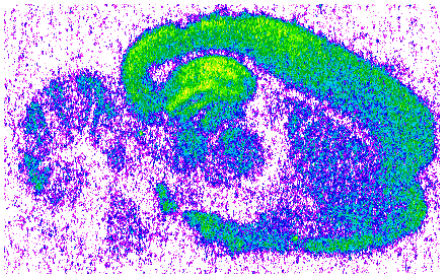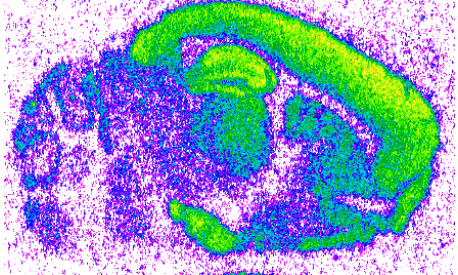

kainate

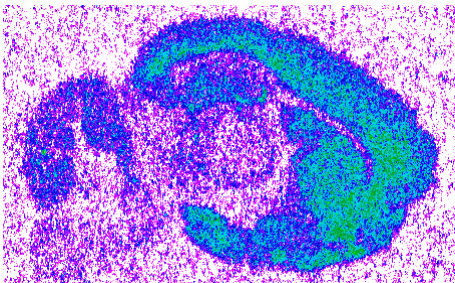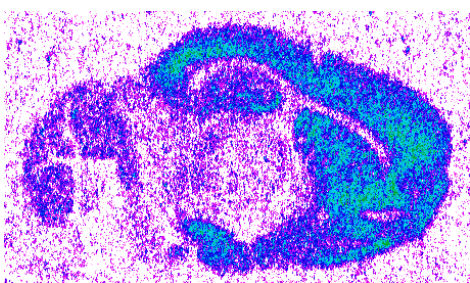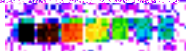

Supplement: FIGURE S1 — The examples of autoradiography binding pictures (sagittal sections) for specific receptor subtypes. Left column: binding to specific receptor subtype in WT animals. Right column: binding to specific receptor subtype in M4 KO animals. First to sixth row: binding to MR, D1-like, D2-like, GABAA, NMDA and kainate receptors, respectively. Bottom: radioactivity standards. [file Data_Sheet_1.PDF]
