# Supplementary material for: Variability in the Drug Response of M4 Muscarinic Receptor Knockout Mice During Day and Night Time
Source: Front Pharmacol. 2019 Mar 18;10:237. doi: 10.3389/fphar.2019.00237 (PMC6431655; doi:10.3389/fphar.2019.00237)
Supplement: TABLE S1 — The autoradiography binding to brain regions for specific receptor subtypes. [file Data_Sheet_2.PDF]

Supplementary Table 1: The autoradiography binding to brain regions for specific receptor subtypes.

	WT		KO		WT		KO	
	**MoCx**				**SSCx**			
	9:00 AM	9:00 PM	9:00 AM	9:00 PM	9:00 AM	9:00 PM	9:00 AM	9:00 PM
**kainate**	8.12 ± 0.53	6.87 ± 0.31	7.96 ± 0.31	5.85 ± 0.21	7.67 ± 0.43	6.61 ± 0.33	7.38 ± 0.65	5.62 ± 0.18
**$GABA_A$**	73.02 ± 2.12	58.80 ± 3.08	75.54 ± 2.77	57.46 ± 2.60	17.58 ± 1.38	19.46 ± 1.15	19.81 ± 1.74	17.70 ± 0.77
**MR**	73.02 ± 2.59	58.80 ± 3.08	75.54 ± 2.77	57.46 ± 2.60	81.09 ± 2.90	56.52 ± 3.17	79.04 ± 2.29	58.40 ± 1.93
**NMDA**	12.63 ± 1.22	12.21 ± 4.71	14.82 ± 4.39	15.41 ± 4.69	11.83 ± 1.25	12.62 ± 4.27	13.41 ± 4.16	15.10 ± 4.38
**D_1-like**	2.71 ± 0.32	2.70 ± 0.43	3.32 ± 0.60	2.75 ± 0.25	N.A.	N.A.	N.A.	N.A.
**D_2-like**	N.A.	N.A.	N.A.	N.A.	N.A.	N.A.	N.A.	N.A.
	**VisCx**				**Hipp**			
	9:00 AM	9:00 PM	9:00 AM	9:00 PM	9:00 AM	9:00 PM	9:00 AM	9:00 PM
**kainate**	8.19 ± 0.15	6.20 ± 0.20	8.02 ± 0.47	6.07 ± 0.26	6.88 ± 0.30	5.55 ± 0.24	7.31 ± 0.46	5.10 ± 0.20
**$GABA_A$**	17.87 ± 1.07	19.04 ± 1.76	18.87 ± 4.32	16.84 ± 1.66	13.85 ± 0.69	14.81 ± 0.60	14.19 ± 3.40	13.29 ± 1.23
**MR**	80.90 ± 3.38	54.58 ± 5.07	79.97 ± 3.21	59.04 ± 2.51	88.94 ± 3.70	81.39 ± 3.25	90.02 ± 2.54	78.71 ± 2.56
**NMDA**	9.96 ± 0.38	11.26 ± 3.44	13.00 ± 4.16	13.46 ± 3.95	13.82 ± 1.00	15.68 ± 1.42	18.37 ± 5.35	18.73 ± 5.59
**D_1-like**	N.A.	N.A.	N.A.	N.A.	N.A.	N.A.	N.A.	N.A.
**D_2-like**	N.A.	N.A.	N.A.	N.A.	N.A.	N.A.	N.A.	N.A.
	**CA1**				**CA3**			
	9:00 AM	9:00 PM	9:00 AM	9:00 PM	9:00 AM	9:00 PM	9:00 AM	9:00 PM
**kainate**	2.93 ± 0.25	2.63 ± 0.13	3.02 ± 0.33	2.36 ± 0.17	6.89 ± 0.22	5.67 ± 0.31	7.16 ± 0.60	5.10 ± 0.20
**$GABA_A$**	15.23 ± 0.77	16.66 ± 0.99	16.44 ± 3.84	15.14 ± 1.39	6.80 ± 0.48	7.64 ± 0.47	7.14 ± 1.79	7.01 ± 0.77
**MR**	107.68 ± 4.26	97.57 ± 3.96	109.92 ± 3.05	98.02 ± 3.18	75.98 ± 2.55	69.36 ± 3.60	78.29 ± 2.47	67.88 ± 3.20
**NMDA**	18.56 ± 1.50	21.64 ± 1.90	23.10 ± 6.75	25.33 ± 7.59	11.63 ± 3.49	11.65 ± 1.63	13.61 ± 4.11	13.77 ± 4.09
**D_1-like**	N.A.	N.A.	N.A.	N.A.	N.A.	N.A.	N.A.	N.A.
**D_2-like**	N.A.	N.A.	N.A.	N.A.	N.A.	N.A.	N.A.	N.A.

	**DG**				**CPu**			
	9:00 AM	9:00 PM	9:00 AM	9:00 PM	9:00 AM	9:00 PM	9:00 AM	9:00 PM
**kainate**	5.35 ± 0.19	4.70 ± 0.19	5.58 ± 0.50	3.72 ± 0.18	7.30 ± 0.37	6.79 ± 0.32	7.36 ± 0.34	6.00 ± 0.14
**$GABA_A$**	15.33 ± 0.84	16.43 ± 0.69	15.63 ± 3.82	14.32 ± 1.30	7.07 ± 1.40	9.82 ± 0.41	8.30 ± 2.07	8.14 ± 0.50
**MR**	85.49 ± 4.49	77.42 ± 3.30	84.36 ± 2.17	71.43 ± 3.08	94.86 ± 4.97	52.60 ± 1.41	94.61 ± 2.09	50.58 ± 1.36
**NMDA**	13.50 ± 0.62	14.69 ± 1.25	17.64 ± 5.14	16.77 ± 4.95	2.98 ± 0.40	2.77 ± 1.37	2.40 ± 0.44	3.05 ± 0.77
**D_1-like**	N.A.	N.A.	N.A.	N.A.	53.65 ± 3.58	52.99 ± 2.63	55.33 ± 2.08	54.26 ± 3.17
**D_2-like**	N.A.	N.A.	N.A.	N.A.	6.98 ± 0.45	6.47 ± 0.33	6.41 ± 0.29	6.38 ± 0.20
	**NAc**				**TH**			
	9:00 AM	9:00 PM	9:00 AM	9:00 PM	9:00 AM	9:00 PM	9:00 AM	9:00 PM
**kainate**	9.37 ± 0.66	8.82 ± 0.26	9.75 ± 0.31	8.01 ± 0.33	1.65 ± 0.17	1.12 ± 0.07	1.48 ± 0.10	1.05 ± 0.09
**$GABA_A$**	9.92 ± 1.15	12.12 ± 0.48	11.57 ± 2.83	11.59 ± 0.72	19.35 ± 0.38	23.30 ± 1.42	21.06 ± 4.85	18.53 ± 1.99
**MR**	108.53 ± 5.56	69.36 ± 2.32	109.54 ± 3.04	67.75 ± 1.37	21.30 ± 1.48	11.97 ± 0.59	20.44 ± 0.71	11.78 ± 0.73
**NMDA**	7.84 ± 3.22	5.87 ± 1.77	8.10 ± 2.57	7.78 ± 2.34	1.42 ± 0.42	3.38 ± 1.32	4.39 ± 1.32	2.90 ± 1.06
**D_1-like**	53.10 ± 3.88	51.62 ± 2.99	57.35 ± 1.19	56.55 ± 2.30	N.A.	N.A.	N.A.	N.A.
**D_2-like**	6.82 ± 0.68	6.18 ± 0.29	6.64 ± 0.29	6.32 ± 0.18	N.A.	N.A.	N.A.	N.A.
	**OT**							
	9:00 AM	9:00 PM	9:00 AM	9:00 PM				
**kainate**	8.60 ± 0.51	7.91 ± 0.46	9.40 ± 0.49	7.23 ± 0.40				
**$GABA_A$**	14.36 ± 0.62	14.58 ± 1.05	14.83 ± 3.35	14.18 ± 1.01				
**MR**	98.49 ± 4.55	51.01 ± 1.66	107.87 ± 3.41	48.00 ± 2.32				
**NMDA**	10.48 ± 3.03	9.70 ± 2.97	12.67 ± 3.79	8.60 ± 2.50				
**D_1-like**	59.60 ± 4.72	57.38 ± 0.84	63.96 ± 0.74	60.69 ± 2.74				
**D_2-like**	6.93 ± 0.42	5.97 ± 0.13	7.00 ± 0.50	6.18 ± 0.18				

Data are expressed as means±S.E.M [nCi/mg]. The specific brain areas are: motor cortex (MOCx), somatosensory cortex (SSCx), visual cortex (VisCx), striatum (caudate-putamen, CPu), nucleus accumbens (NAc), thalamus (TH), hippocampus (Hipp) and its specific areas CA1, CA3 and dentate gyrus (DG), olfactory tubercle (OT). N.A. non-analyzed, i.e. the density was apparently the same in these brain areas. For differences see figures in the main manuscript.
